# Supplementary material for: Pyrimidine sufficiency is required for Sae two-component system signaling in Staphylococcus aureus
Source: J Bacteriol. 2025 Jul 21;207(8):e00115-25. doi: 10.1128/jb.00115-25 (PMC12369341; doi:10.1128/jb.00115-25)
Supplement: Supplemental materials — Supplemental figures, tables, and references. [file jb.00115-25-s0001.docx]

Pyrimidine sufficiency is required for Sae two-component system signaling in *Staphylococcus aureus*

Dennis A. DiMaggio, Jr., Won-Sik Yeo, and Shaun R. Brinsmade

**Supplemental Materials**

**Figure S1: HNP1 activates the Sae TCS during pyrimidine limitation**. The indicated LAC strains were grown in TSB for 6 h and incubated in the presence or absence of HNP1. *sae*P1-*gfp* promoter activity was measured after 2 h and RFU were plotted. Data are plotted as mean ± SEM from three independent experiments. ***p<0.001, **p<0.01; unpaired Welch’s *t*-test with Holm-Šídák's multiple comparisons test. nd, below detectible limit.

**Figure S2: *S. aureus* requires *de novo* synthesis of pyrimidines during laboratory growth. (A)** Wild type (WT) and ∆*pyrE* mutant cells were grown in tryptic soy broth (TSB) ± uracil supplementation (Ura; 200 µM). Growth was monitored and plotted as increase in absorbance at 600 nm (OD_600_) over time. **(B)** Extended growth data for WT and *∆pyrE* mutant cells grown in TSB. **(C)** Cell enumeration data for indicated timepoints in panel B are plotted as colony forming units per ml of culture (CFU/ml) over time. **(D)** USA300 LAC **(**WT), *pyrE::Tn, ∆pyrE, ∆pyrD,* and *∆sae* mutant cells were streaked out on TSA plates. The primary streak of the ∆*pyrE* mutant is expanded in inset to show accumulation of white, crystalline material. In panels A-C, data are plotted as mean ± SEM of three independent biological replicates. Error bars for panel A and B are plotted for all time points, in most cases they are too small to be seen. For panel C, bar indicates mean ± SEM. ****p<0.0001, Two-way ANOVA with Tukey’s post-test.

**Figure S3. The Sae TCS defect can be rescued by cytidine supplementation and by genetic complementation.** Measurement of *sae*P1-*gfp* promoter activity in stationary phase cultures (16 h) grown in TSB showing the effect of **(A)** chemical complementation with cytidine (Cyd; 200 µM) and **(B)** genetic complementation of the ∆*pyrD* mutant for activation of the Sae TCS. *+pyrD^+^*, *pyrD* complementation vector; EV, empty vector. Data are plotted as mean ± SEM of ≥ 3 independent experiments. ****p<0.0001, ***p<0.001, One-way ANOVA with Tukey’s post-test. ns, not significant.

**Figure S4: Overproduction of LTAs is not responsible for the Sae TCS defect in *∆pyrE* mutant cells.**The indicated strains were grown to stationary phase (16 h) in TSB. Cells were then pelleted and subjected to protein or LTAs extraction as described in *Materials and Methods*. **(A)** Representative LTA Western blot probing with monoclonal anti-LTA antibodies. **(B)** Densitometry analysis of LTA Western blots. LTA values were normalized by dividing the average intensity of the entire LTA profile by the total number of colony forming units in each sample. **(C)** Top: SDS-PAGE and Phos-tag Western blots probing for SaeR protein. LC, loading control. Bottom: Densitometry analysis of SaeR Western blots quantifying SaeR~P abundance relative to total SaeR signal in the blot per sample. EV, empty vector; +*mspA*^+^, *mspA* complementation vector. Data are plotted as mean ± SEM of three biological replicates. ****p<0.0001, ***p<0.001, **p<0.01, one-way ANOVA with Tukey’s post-test. ns, not significant; nd, below detectible limit.

**Figure S5: Overexpression of *mspA* does not alter SaeRS TCS activity in ∆*pyrE* mutant cells.** The indicated strains were grown to stationary phase (16 h) in TSB with or without the addition of anhydrotetracycline (aTc; 25 ng/ml). Cells were then normalized by OD_600_ and cell lysates were subjected to Western blot analysis. Top: SDS-PAGE and Phos-tag Western blots probing for SaeR protein. LC, loading control. Bottom: Densitometry analysis of SaeR Western blots quantifying SaeR~P abundance relative to total SaeR signal in the blot per sample. EV, empty vector; +*mspA*^+^, *mspA* complementation vector. Inset plot on left shows simple comparison of %SaeR~P in the isogenic parental strains, while the right inset plot highlights the ∆*pyrE* mutants harboring the empty vector or the *mspA^+^* overexpression construct ± aTc induction. All data are plotted as mean ± SEM of at least three biological replicates. ****p<0.0001, ***p<0.001, **p<0.01, *p<0.05, one-way ANOVA with Tukey’s post-test. ns, not significant; nd, below detectible limit.

**Figure S6: Staphyloxanthin production is increased in ∆*pyrE* mutant cells but does not affect Sae activity.** Indicated strains were grown to stationary phase (24 h) in TSB. Cells were then pelleted and washed, and carotenoid pigment was extracted using hot methanol as described in *Materials and Methods*. **(A)** Carotenoid values were normalized by dividing the A_465_ value by the total number of colony forming units in each sample. **(B)** Indicated strains were grown to stationary phase (16 h) and *sae*P1-*gfp* promoter activity was measured after washing cells and resuspending in PBS. Data are plotted as mean ± SEM of three biological replicates. ****p<0.0001, ***p<0.001, *p<0.05, one-way ANOVA with Tukey’s post-test. ns, not significant.

**Figure S7: Pyrimidine-limited cells are sensitized to fatty acid addition.**

Indicated strains carrying *sae*P1*-gfp* reporter fusions were grown overnight (16 h) with the addition of **(A)** BSA at 10 and 20 mg/ml or **(B)** BSA 10 mg/ml conjugated with 15:0 *anteiso*-fatty acid (FA) and RFU were measured. <, fluorescence was undetected relative to blank. For both panels, bars represent mean ± SEM of three biological replicates. ****p<0.0001, ***p<0.001, **p<0.01, one-way ANOVA with Tukey’s post-test. ns, not significant.

**Supplemental Tables**

**Data Table S1. Transcriptomic analysis of *∆pyrE* mutant cells during pyrimidine limitation.** Transcriptomic profiling of WT and *∆pyrE* mutant cells grown to pyrimidine limitation (8) h in TSB. Raw reads were trimmed using Trimmomatic, trimmed reads were aligned and analyzed based on the reference genome (USA300 FPR3757; NC007793) using Rockhopper. *p-*values were determined using a Negative Binomial distribution and adjusted *p*-values were determined using the Benjamin Hochberg procedure built into the script. Up-regulated and down-regulated genes based on Log_2_ fold change ≥ 1 or ≤-1 and *p*<0.05 *∆pyrE*/WT are listed.

**Data Table S2. KEGG analysis reveals shifts in many different biochemical pathways in the *∆pyrE* mutant during pyrimidine limitation.** RNA-sequencing data from Table S1 were re-analyzed in R using the KEGGREST package to assess changes in transcripts of genes involved in various biochemical pathways, using the USA300 FPR3757 KEGG genome as reference (saa; KEGG) (1, 2). Pathway code; Code for respective pathway derived from KEGG; Annotated Genes; genes annotated to be involved in the listed biochemical pathway; DEG, Differentially Expressed Gene; Up, Up-regulated genes; Down, Down-regulated genes; Pathway Name, Pathway name annotated in KEGG.

**Table S1 Minimum Inhibitory Concentrations (MIC) (µg/ml; % for Congo Red) of compounds targeting cell envelope biosynthesis**

| **Strain** | **Oxacillin** | **Tunicamycin** | **Fosfomycin** | **Congo Red** |
| --- | --- | --- | --- | --- |
| WT | 128 | 256 | ≥256 | 0.25% |
| ∆*pyrE* | 128 | 256 | 128-256 | 0.0625-0.03125% |
|  |  |  |  |  |

WT and *∆pyrE* mutant cells were grown in the presence of chemical inhibitors for various steps in cell envelope biosynthesis using the CLSI microdilution method (Oxacillin-Penicillin binding protein; Tunicamycin-Wall Teichoic Acids; Fosfomycin-Cell Wall; Congo Red- Lipoteichoic Acids) (3). Data represent the average MIC of three independent biological replicates.

**Table S2. Strains used in this study.**

| Strain | Genotype or description | Source or reference^§^ |
| --- | --- | --- |
| NE1444 | USA300 JE2 *crtM*::φNΣ | (4) |
| RN4220 | Restriction-deficient, highly transformable | (5) |
| SRB687 | USA300 LAC CA-MRSA Em^S^ (AH1263) (“LAC”) | A.R. Horswill |
| SRB1188 | LAC ∆*saePQRS* | (6) |
| SRB1395 | LAC Δ*saePQRS* / pCL55 [P*sae*P3-*saeRS*] (Cm^R^) | (6) |
| SRB1412 | LAC / pCL55 [*sae*P1*-gfp*] (Cm^R^) | (6) |
| SRB1487 | RN4220 / pLL2787 (Cm^R^) | (7) |
| SRB1516 | RN4220 / pLL39 (Tet^R^) | (7) |
| SRB1712 | LAC *lpdA*::φNΣ *mbcS1* (Em^R^) | (8) |
| SRB1786 | RN4220 / pMA15 (Cm^R^; Kan^R^) | (9) |
| SRB1787 | LAC / pOS-*sae*P1-*gfp* (Cm^R^) |  |
| SRB1788 | LAC / pYJ-*sae*P1-*gfp* (Em^R^) |  |
| SRB1790 | LAC / pMA15 (Cm^R^; Kan^R^) |  |
| SRB1796 | LAC *∆saePQRS* / pOS-*sae*P1-*gfp* (Cm^R^) |  |
| SRB1806 | LAC *∆saePQRS* / pYJ-*sae*P1-*gfp* (Em^R^) |  |
| SRB2339 | LAC ∆*saePQRS* / pCL55 [EV] (Cm^R^) |  |
| SRB2355 | LAC *pyrE::kan^R^* |  |
| SRB2357 | LAC *pyrE::kan^R^* / pYJ-*sae*P1-*gfp* (Em^R^) |  |
| SRB2370 | LAC ∆*saePQRS* / pCL55 [*sae*P1-*gfp*] (Cm^R^) |  |
| SRB2728 | DH5α / pDD02 (Ap^R^) |  |
| SRB2729 | DH5α / pDD03 (Ap^R^) |  |
| SRB2738 | RN4220 / pDD02 (Cm^R^) |  |
| SRB2739 | RN4220 / pDD03 (Cm^R^) |  |
| SRB2748 | LAC / pDD02 (Cm^R^) |  |
| SRB2749 | LAC / pDD03 (Cm^R^) |  |
| SRB2797 | LAC ∆*pyrE* |  |
| SRB2799 | LAC ∆*pyrD* |  |
| SRB2808 | LAC *∆pyrE* / pYJ-*sae*P1-*gfp* (Em^R^) |  |
| SRB2810 | LAC *∆pyrD* / pYJ-*sae*P1-*gfp* (Em^R^) |  |
| SRB2825 | RN4220 / pLL2787; pDD05 (Cm^R^; Tet^R^) |  |
| SRB2868 | LAC *∆saePQRS* / pDD02 (Cm^R^) |  |
| SRB2869 | LAC *∆saePQRS* / pDD03 (Cm^R^) |  |
| SRB2891 | LAC *lpdA::*φNΣ *mbcS1* / pOS-*sae*P1-*gfp* (Em^R^; Cm^R^) |  |
| SRB2907 | LAC *∆pyrD* / pDD05 (Tet^R^) |  |
| SRB2908 | LAC ∆*saePQRS* ∆*pyrE* |  |
| SRB2909 | LAC ∆*saePQRS* ∆*pyrD* |  |
| SRB2932 | LAC ∆*saePQRS* ∆*pyrE* / pCL55 [EV] (Cm^R^) |  |
| SRB2933 | LAC ∆*saePQRS* ∆*pyrE* / pCL55 [P*sae*P3-*saeRS*] (Cm^R^) |  |
| SRB3034 | LAC ∆*pyrD* / pLL39 (Tet^R^) |  |
| SRB3036 | LAC ∆*saePQRS ∆pyrE* / pYJ-*sae*P1-*gfp* (Em^R^) |  |
| SRB3059 | DH5α / pDD10 (Cm^R^) |  |
| SRB3062 | RN4220 / pDD10 (Cm^R^) |  |
| SRB3066 | LAC / pDD10 (Cm^R^) |  |
| SRB3069 | LAC *∆pyrD* / pDD05; pYJ-*sae*P1-*gfp* (Tet^R^; Em^R^) |  |
| SRB3072 | LAC *∆pyrD* / pDD05; pYJ-*sae*P1-*gfp* (Tet^R^; Em^R^) |  |
| SRB3093 | LAC ∆*pyrC* |  |
| SRB3116 | LAC *∆pyrC* / pYJ-P1-GFP (Em^R^) |  |
| SRB3120 | LAC *crtM::* φNΣ (Em^R^) |  |
| SRB3128 | LAC ∆*pyrE crtM::* φNΣ (Em^R^) |  |
| SRB3131 | LAC ∆*pyrE* / pOS-*sae*P1-*gfp* (Cm^R^) |  |
| SRB3135 | LAC *∆pyrE ∆saePQRS* / pCL55 [EV]; pYJ-*sae*P1-*gfp* (Cm^R^; Em^R^) |  |
| SRB3137 | LAC *∆pyrE ∆saePQRS* / pCL55 [P*sae*P3*-saeRS*]; pYJ-*sae*P1-*gfp* (Cm^R^; Em^R^) |  |
| SRB3175 | *crtM::* φNΣ / pCL55 [*saeP1-sGFP*] (Em^R^; Cm^R^) |  |
| SRB3176 | LAC *∆pyrE* / pCL55 [*saeP1-sGFP*] (Cm^R^) |  |
| SRB3177 | LAC ∆*pyrE crtM::* φNΣ / pCL55 [*sae*P1*-gfp*] (Em^R^; Cm^R^) |  |
| SRB3402 | JE2 *mspA::Tn* (Em^R^) | (10, 11) |
| SRB3403 | JE2 *mspA::Tn* / pRMC2 (EV) (Em^R^; Cm^R^) | (10, 11) |
| SRB3404 | JE2 *mspA::Tn* / pRMC2-*mspA*^+^ (Em^R^; Cm^R^) | (10, 11) |
| SRB3420 | LAC *mspA::Tn* |  |
| SRB3508 | LAC *mspA::Tn*/ pRMC2-EV (Em^R^; Cm^R^; Tet^R^) |  |
| SRB3509 | LAC *mspA::Tn* / pRMC2-*mspA*^+^ (Em^R^; Cm^R^; Tet^R^) |  |
| SRB3510 | LAC *∆pyrE mspA::Tn*/ pRMC2-EV (Em^R^; Cm^R^; Tet^R^) |  |
| SRB3511 | LAC *∆pyrE mspA::Tn* / pRMC2-*mspA*^+^ (Em^R^; Cm^R^; Tet^R^) |  |
| SRB3584 | LAC ∆*saePQRS* / pCL55 [P*sae*P3*-saeRSc*] (Cm^R^) |  |
| SRB3585 | LAC ∆*pyrE* ∆*saePQRS* / pCL55 [P*sae*P3*-saeRSc*] (Cm^R^) |  |
| SRB3608 | LAC ∆*saePQRS* / pCL55 [P*sae*P3*-saeRSc*]; pYJ-*sae*P1-*gfp* (Cm^R^, Em^R^) |  |
| SRB3609 | LAC ∆*pyrE* ∆*saePQRS* / pCL55 [P*sae*P3*-saeRSc*]; pYJ-*sae*P1-*gfp* (Cm^R^, Em^R^) |  |
| SRB3931 | LAC / pRMC2-EV (Cm^R^; Tet^R^) |  |
| SRB3932 | LAC / pRMC2-*mspA*^+^ (Cm^R^; Tet^R^) |  |
| SRB3933 | LAC ∆*pyrE* / pRMC2-EV (Cm^R^; Tet^R^) |  |
| SRB3934 | LAC ∆*pyrE* / pRMC2-*mspA*^+^ (Cm^R^; Tet^R^) |  |
| §Unless otherwise indicated, the strains were constructed during this study. | | |

**Table S3. Oligonucleotides used in this study.**

| Oligonucleotides | Sequence (5’ 🡪 3’) | Usage |
| --- | --- | --- |
| oCS033 | CTGGGGAATTGGGAATTGAT | pLL39 insert sequencing forward |
| oDD032 | CGGTAGACCGATTACACAAAG | *pyrE* sequencing forward |
| oDD033 | GCAGCCCCTGATATTAAAC | *pyrE* sequencing reverse |
| oDD040 | CGTTATTCAAACTTGCGTC | *pyrD* sequencing forward |
| oDD041 | CTTTAGTCATAACAAACGCCTC | *pyrD* sequencing reverse |
| oDD060 | ATAAGTATCATTTATTAACTTTCCATTGGTACCGGTTCCG | pDD02, Gibson construction 500 bp upstream of *pyrE* including ATG forward |
| oDD061 | GTATTAGGAATATCATGCAGATCTCTAGCTAGCTAGCAGCTGC | pDD02, Gibson construction 500 bp upstream of *pyrE* including ATG reverse |
| oDD062 | GCTGCTAGCTAGCTAGAGATCTGCATGATATTCCTAATACAGTTGGTAAGGC | pDD02, Gibson construction 500 bp downstream of *pyrE* including TAA forward |
| oDD063 | TCCTTCTAGTGTCTATATTACATTATACTAACCAACTTTCTTTAATTTTATGATAACTTTCGACTG | pDD02, Gibson construction 500 bp downstream of *pyrE* including TAA reverse |
| oDD064 | AAAGTTGGTTAGTATAATGTAATATAGACACTAGAAGGAGGAATTCAACAAATGAATGAC | pDD02, Gibson construction to amplify pKOR1_mcs vector backbone forward |
| oDD065 | CGGAACCGGTACCAATGGATTGTTAATAAATGATACTTATCATAGGTATTAATGTTTTGACTTATTGT | pDD02, Gibson construction to amplify pKOR1_mcs vector backbone reverse |
| oDD066 | TTTACGATTATATAAATAACATCCATTGGTACCGGTTCCG | pDD03, Gibson construction to amplify pKOR1_mcs vector backbone forward |
| oDD067 | GGGCTTCAGGGAGTCAACGCATCTCTAGCTAGCTAGCAGCTGC | pDD03, Gibson construction to amplify pKOR1_mcs vector backbone reverse |
| oDD068 | GCTGCTAGCTAGCTAGAGATGCGTTGACTCCCTGAAGCC | pDD03, Gibson construction 500 bp upstream of *pyrD* including ATG forward |
| oDD069 | TATTTGACTATGTTGGATTACATGAGTATGCTCCTATTTCATTATATTTGAGGC | pDD03, Gibson construction 500 bp upstream of *pyrD* including ATG reverse |
| oDD070 | ATAGGAGCATACTCATGTAATCCAACATAGTCAAATAATACTTTAAAACTTGCTGAATCG | pDD03, Gibson construction 500 bp downstream of *pyrD* including TAA forward |
| oDD071 | CGGAACCGGTACCAATGGATGTTATTTATATAATCGTAAATATTGTAGATATAATGAATAACTGGATCC | pDD03, Gibson construction 500 bp downstream of *pyrD* including TAA reverse |
| oDD072 | CACACAGGAAACAGCTATGAC | To confirm pKOR1_mcs deletion constructs |
| oDD073 | CTGCCCCGTTAGTTGAAG | To confirm pKOR1_mcs deletion constructs |
| oDD078 | TCTAGAGGATCCCCTATACTATAATATTGCCTAATTAGGATGATTTAATCTTAAAAACGT | To amplify *pyrD* insert with SacI cutting site |
| oDD079 | CGAATTCGAGCTCGGTACCCTTAGGCATCTAGTCCTATAATATCACTGACATTGT | To amplify *pyrD* insert with BamHI cutting site |
| oDD083 | CAGTGAATGGGGGTAAATGG | pLL39 insert sequencing reverse |
| oDD132 | TTCGACTAAAACACCGTATGATCCATTGGTACCGGTTCCG | pDD10, Gibson construction to amplify pKOR1_mcs vector backbone forward |
| oDD133 | TCTTCATATATCGTCATTAAATCTCTAGCTAGCTAGCAGCTGC | pDD10, Gibson construction to amplify pKOR1_mcs vector backbone reverse |
| oDD134 | GCTGCTAGCTAGCTAGAGATTTAATGACGATATATGAAGAATATGGATATTTTGAAGGC | pDD10, Gibson construction 500 bp upstream of *pyrC* including ATG forward |
| oDD135 | AACGTTTGCTTTGCATATTACATCATTTTCGTCCCCTTACCTATTTTAATAATTCA | pDD10, Gibson construction 500 bp upstream of *pyrC* including ATG reverse |
| oDD136 | GTAAGGGGACGAAAATGATGTAATATGCAAAGCAAACGTTATCTAGTGTTAGAAGACGGT | pDD10, Gibson construction 500 bp downstream of *pyrC* including TAA forward |
| oDD137 | CGGAACCGGTACCAATGGATCATACGGTGTTTTAGTCGAAACGATTTCTAC | pDD10, Gibson construction 500 bp downstream of *pyrC* including TAA reverse |
| oDD143 | GCAAATGGAAAATGGCG | *pyrC* sequencing forward |
| oDD144 | CTAAAATTACTTGGATGCGC | *pyrC* sequencing reverse |
| oWY41 | AGTGTGGTTGTTTTTGTTGGAAGTGTG | inverse PCR for Tn Kan from pMA15 |
| oWY42 | GGTGGTGTGGTTTTGTTGGTTG | inverse PCR for Tn Kan from pMA15 |

**Table S4. Plasmids used in this study.**

| Plasmid | Description | Source or reference^§^ |
| --- | --- | --- |
| pMA15 | Temperature sensitive vector used for random transposon mutagenesis, harbors hyperactive *Himar1 mariner* transposase C9 driven under the *sae*P3 promoter. Kanamycin resistance cassette is located between two terminal inverted repeats, Kan^R^, Cm^R^ | (9) |
| pCL55 | Empty vector control, Ap^R^, Cm^R^ | (7) |
| pCL55-saeRS | Integrated vector containing *saeRS* driven under constitutive P3 promoter, Ap^R^, Cm^R^ | (12) |
| pCL55-saeRSc | Integrated vector containing *saeRS;* only expressing catalytically active domain of SaeS driven under constitutive P3 promoter, Ap^R^, Cm^R^ | (12) |
| pCL55-*sae*P1-*gfp* | Integrated vector carrying the *sae*P1 promoter driving expression of GFP, Ap^R^, Cm^R^ | (6) |
| pLL2787 | *S. aureus* single integration vector helper plasmid Cm^R^ | (7) |
| pLL39 | *S. aureus* single integration vector Tet^R^ | (7) |
| pYJ-*sae*P1-*gfp* | pYJ335-GFP carrying the *sae*P1 promoter | (13) |
| pOS-*sae*P1-*gfp* | pOS1 carrying the *sae*P1 promoter driving expression of GFP | (14) |
| pKOR1 | Allelic replacement plasmid with inducible counter-selection. | (15) |
| pDD02 | pKOR1 with *pyrE* allelic exchange construct cloned in via Gibson assembly, Ap^R^, Cm^R^ |  |
| pDD03 | pKOR1 with *pyrD* allelic exchange construct cloned in via Gibson assembly, Ap^R^, Cm^R^ |  |
| pDD05 | pLL39 with *pyrD* expressed under its native promoter |  |
| pDD10 | pKOR1 with *pyrC* allelic exchange construct cloned in via Gibson assembly, Ap^R^, Cm^R^ |  |
| pRMC2 | Tetracycline-inducible expression vector for *S. aureus*; Tet^R^,Cm^R^ | (16) |
| pRMC2-*mspA^+^* | pRMC2 with *mspA* gene expressed under tet inducible promoter; Tet^R^,Cm^R^ | (10) |
| ^§^ Unless otherwise indicated, the plasmids were constructed during this study. | | |

Bibliography and References Cited:

1. Tenenbaum D. 2016. KEGGREST: Client-side REST access to KEGG. R package version 1.

2. Kanehisa M, Goto S. 2000. KEGG: kyoto encyclopedia of genes and genomes. Nucleic Acids Res 28:27-30.

3. CLSI. 2018. Methods for dilution antimicrobial susceptibility tests for bacteria that grow aerobically, p 27-50, CLSI standard M07-A11, 11 ed.

4. Fey PD, Endres JL, Yajjala VK, Widhelm TJ, Boissy RJ, Bose JL, Bayles KW. 2013. A genetic resource for rapid and comprehensive phenotype screening of nonessential *Staphylococcus aureus* genes. MBio 4:e00537-12.

5. Kreiswirth BN, Löfdahl S, Betley MJ, O'Reilly M, Schlievert PM, Bergdoll MS, Novick RP. 1983. The toxic shock syndrome exotoxin structural gene is not detectably transmitted by a prophage. Nature 305:709-712.

6. Collins MM, Behera RK, Pallister KB, Evans TJ, Burroughs O, Flack C, Guerra FE, Pullman W, Cone B, Dankoff JG, Nygaard TK, Brinsmade SR, JM. V. 2020. The Accessory Gene *saeP* of the SaeR/S Two-Component Gene Regulatory System Impacts *Staphylococcus aureus* Virulence During Neutrophil Interaction. Front Microbiol 11:561.

7. Luong TT, Lee CY. 2007. Improved single-copy integration vectors for Staphylococcus aureus. J Microbiol Methods 70:186-90.

8. Pendleton A, Yeo WS, Alqahtani S, DiMaggio Jr DA, Stone CJ, Li Z, Singh VK, Montgomery CP, Bae T, Brinsmade SR. 2022. Regulation of the Sae Two-Component System by Branched-Chain Fatty Acids in *Staphylococcus aureus*. mBio 13:e0147222.

9. Zhao N, Wang Y, Liu J, Yang Z, Jian Y, Wang H, Ahmed M, Li M, Bae T, Liu Q. 2022. Molybdopterin biosynthesis pathway contributes to the regulation of SaeRS two-component system by ClpP in Staphylococcus aureus. Virulence 13:727-739.

10. Duggan S, Laabei M, Alnahari AA, O'Brien EC, Lacey KA, Bacon L, Heesom K, Fu CL, Otto M, Skaar E, McLoughlin RM, Massey RC. 2020. A Small Membrane Stabilizing Protein Critical to the Pathogenicity of Staphylococcus aureus. Infect Immun 88.

11. Bonini D, Duggan S, Alnahari A, Brignoli T, Strahl H, Massey RC. 2024. Lipoteichoic acid biosynthesis by Staphylococcus aureus is controlled by the MspA protein. mBio 15:e0151224.

12. Liu Q, Cho H, Yeo WS, Bae T. 2015. The extracytoplasmic linker peptide of the sensor protein SaeS tunes the kinase activity required for staphylococcal virulence in response to host signals. PLoS Pathog 11:e1004799.

13. Yeo WS, Arya R, Kim KK, Jeong H, Cho KH, Bae T. 2018. The FDA-approved anti-cancer drugs, streptozotocin and floxuridine, reduce the virulence of Staphylococcus aureus. Sci Rep 8:2521.

14. Alqahtani S, DiMaggio Jr DA, Brinsmade SR. 2024. CodY controls the SaeR/S two-component system by modulating branched-chain fatty acid synthesis in *Staphylococcus aureus*. J Bacteriol In press.

15. Bae T, Schneewind O. 2006. Allelic replacement in Staphylococcus aureus with inducible counter-selection. Plasmid 55:58-63.

16. Corrigan RM, Foster TJ. 2009. An improved tetracycline-inducible expression vector for Staphylococcus aureus. Plasmid 61:126-9.
